# Evaluating the environmental sustainability of AI in radiology: a systematic review of current practice

**DOI:** 10.1136/bmjdhai-2025-000073

**Published:** 2025-10-03

**Authors:** Rachel M Thomson, Jesus Perdomo-Lampignano, Euan Fisher, Cindy Wati, Gowsikan Jeyakumar, Sean Duncan, David J Lowe

**Affiliations:** 1 University of Glasgow, Glasgow, UK; 2 NHS Education for Scotland, Edinburgh, UK; 3 NHS Greater Glasgow & Clyde, Glasgow, UK; 4 University of Central Lancashire, Preston, UK

**Keywords:** artificial intelligence, health equity, implementation science, machine learning

## Abstract

**Objective:**

Data-heavy and energy-heavy artificial intelligence (AI) technologies are increasingly being applied in healthcare, particularly for clinical imaging, often without consideration of their environmental impacts. We aimed to assess current practice in considering and evaluating environmental sustainability (ES) impacts of AI-enabled clinical pathways in radiology.

**Methods and analysis:**

We searched MEDLINE and Embase on 5 November 2024 for quantitative clinical radiology studies which used a machine learning approach to aid in radiological diagnosis or intervention and discussed its ES impacts. We included peer-reviewed, English language studies published from 2015 onwards. Our primary outcome was any quantitative reporting of ES impacts, and our secondary outcome was any within-text qualitative discussion of ES impacts. For quantitative outcomes, we conducted synthesis without meta-analysis based on effect direction and size, with our secondary outcome synthesised narratively.

**Results:**

Of 4449 records screened, 18 met our inclusion criteria. Six (33.33%) reported quantitative ES outcomes and 15 (83.33%) included qualitative discussion of ES. When applied to the same tasks, algorithms designed to be ‘lightweight’ demonstrated from 2.19 to 17.15 times reduction in carbon emissions (median 7.81, 16 datapoints) and from 1.60 to 751.62 times reduction in energy consumption (median 3.22, 16 datapoints) compared with state-of-the-art alternatives, while maintaining similar or improved clinical performance. No quantitative studies compared ES outcomes for an AI-enabled pathway versus standard-of-care, and 75.00% of studies reporting only on our secondary outcome included just a single sentence on sustainability.

**Conclusion:**

Despite increasing concern about the climate impacts of AI, environmental outcomes are rarely considered within evaluations of AI technologies in clinical radiology. However, there are approaches available with smaller carbon footprints. To meet their stated aims on sustainability, funders and governance bodies should consider how to promote integration of environmental impact assessment into AI health research and evaluation.

**PROSPERO registration number:**

CRD42024601818.

**WHAT IS ALREADY KNOWN ON THIS TOPIC**

- Artificial intelligence (AI) technologies are increasingly used in healthcare, with higher energy and data demands than traditional digital tools; however, environmental sustainability is rarely considered in evaluations of AI-enhanced clinical pathways.

**WHAT THIS STUDY ADDS**

- This is the first systematic review to examine how environmental sustainability is assessed in healthcare evaluations of AI, focusing on clinical radiology.
- Very few studies quantitatively evaluated environmental impacts, and these were limited to AI tools specifically designed to be more resource-efficient.
- These studies all reported a reduced environmental impact compared with state-of-the-art competitors—on average, an eight times reduction in carbon emissions and three times reduction in energy consumption—while achieving similar or improved clinical performance.

**HOW THIS STUDY MIGHT AFFECT RESEARCH, PRACTICE OR POLICY**

- Environmental impacts of AI are largely overlooked in current healthcare evaluations, although some tools do have smaller carbon footprints.
- Incorporating sustainability metrics into AI assessments is essential to support responsible innovation and avoid unintended contributions to the climate crisis.

## Introduction

The integration of artificial intelligence (AI) technologies into healthcare has increased rapidly in the last decade with the emergence of powerful deep learning algorithms that can reliably perform complex tasks, particularly in image recognition.^1^ While the theory required to facilitate these advances has existed for decades, recent progress has been driven by increasing availability and decreasing cost of two key commodities: ‘big data’ to train AI models, and sufficient computational power to process and store such data.^2^ These data-intensive and energy-intensive AI algorithms are now being deployed throughout clinical pathways, working on tasks ranging from segmentation of tumours and nodules to clinical risk prediction.^3^ Radiology as a specialty has been a particularly early adopter of AI, producing around a third of all published clinical AI research.^4^

The myriad potential applications mean AI is often touted as a panacea for productivity and efficiency, but the environmental impacts of these technologies are rarely considered in clinical practice.^5 6^ This is despite there being considerable cause for concern^7^: global AI energy demand is projected to exceed that of a country the size of Belgium by 2026,^8^ and the data centres required for model development have significant and increasing impacts on material environments, water consumption, mineral extraction and e-waste disposal.^6^ However, it is worth noting that any efficiencies resulting from AI do have potential to reduce carbon emissions in clinical pathways compared with usual practice, which must be adequately taken into account when considering their overall environmental impact.^9^

Given that healthcare systems will be responsible for managing the increased care burden resulting from the health impacts of the climate crisis, while being major emitters of greenhouse gases themselves, there is a strong argument that clinicians should have a role in the climate response.^10^ One significant way in which clinicians can influence healthcare-related emissions is through involvement in decision-making on sustainable healthcare provision and procurement.^10 11^ However, to facilitate the inclusion of sustainability as a factor in decision-making on adoption and deployment of novel technologies like AI in healthcare, their environmental impacts (both positive and negative) must be transparently measured and reported on.^12^

A recent scoping review suggests environmental assessment remains poorly integrated into most forms of health technology assessment (HTA),^13^ potentially due to the associated technical and resource challenges.^14^ Despite environmental sustainability being a known issue in AI and data-driven research, progress here is particularly slow, with little engagement on the issues of environmental sustainability at all.^6 7^ To our knowledge, there are no existing systematic reviews on this topic specific to AI, and no available guidance on how to select, measure or evaluate sustainable AI software.^5^ We therefore aimed to assess current practice in considering and evaluating the potential or actual environmental sustainability impacts of AI-enabled clinical pathways, focusing on radiology to capitalise on the relative maturity of AI research within this field, and its increasing prioritisation of sustainability.^4 15 16^

## Methods

We conducted a methodological systematic review, pre-registering our protocol with PROSPERO (CRD42024601818). Reporting follows the Preferred Reporting Items for Systematic Reviews and Meta-Analyses (PRISMA) and Synthesis Without Meta-analysis (SWiM) in Systematic Reviews guidance.^17 18^ The minimal protocol deviations are reported in online supplemental appendix A.

10.1136/bmjdhai-2025-000073.supp1 Supplementary data

### Literature search

Searches of peer-reviewed literature were conducted in MEDLINE and Embase on 5 November 2024. To ensure inclusion of studies that were broadly representative of current practice, we designed our search strategy to be in line with that of Kelly *et al* in their 2022 systematic review, which aimed to synthesise the body of evidence on AI use in radiology at that point in time.^3^ Searches included terms for AI combined with the AND operator for terms relevant to radiology AND sustainability (full search strategy in online supplemental appendix B).

#### Inclusion criteria

The relevant population of interest was clinical radiological papers (ie, describing the use of imaging techniques to diagnose, treat or monitor diseases and injuries), which were hospital-based, focused on human participants and published from 2015 onwards. Radiology in this context includes all radiographic, computed tomography (CT), magnetic resonance (MR), ultrasound or nuclear medicine/molecular or hybrid imaging techniques. There were no restrictions on the type of participants in the included studies. The intervention of interest was any machine learning tool used to aid care of a patient’s radiological diagnosis or intervention, which aimed to complete a segmentation, identification, classification or prediction task using computer vision techniques. A comparator group was not required for inclusion.

Our primary outcome group of interest was any quantification or measurement of variables related to environmental sustainability. Specific measures prespecified in the protocol to be included in our primary outcome group were carbon dioxide emissions, other measures of energy usage, water usage, impacts on the material environment, mineral extraction or usage, and waste generation and disposal. Our secondary outcome group of interest was any acknowledgement of or reference to potential environmental sustainability impacts of the intervention within the text of the study or the supplementary materials. This included any qualitative discussion of the following terms or topics, even if not formally quantified: environmental sustainability, climate, carbon dioxide emissions, energy, water and minerals usage, impacts on material environment, and waste generation and disposal.

We included all quantitative, peer-reviewed study types that met our population criteria, including conference abstracts, to ensure inclusion of the most up-to-date AI research.

#### Exclusion criteria

We excluded non-English papers and grey literature, that is, literature which was not formally published in books/journals or peer-reviewed (including preprints). In keeping with Kelly *et al*,^3^ we also excluded non-human, laboratory and phantom-based studies; studies of functional MRI; studies solely for use in radiation therapy; radiomics studies and studies focusing on texture analysis or identification of imaging biomarkers; connectomics and quality assessment studies; image processing and registration studies; and image quality studies.

### Study selection

References were de-duplicated in EndNote 21 and imported to Covidence for screening. All title/abstracts and full texts were independently screened by RMT and a second reviewer (JPL, EF, GJ or CW), with conflicts resolved by consensus and/or discussion with a third reviewer (SD). Reviewer topic/specialty expertise included radiology, public health, oncology and digital innovation in healthcare, with reviewers ranging in stage from medical student (n=1) to senior specialty trainee with ≥3 years of specialty training (n=5).

Relevant commentary articles and reviews captured by the searches were excluded at title and abstract stage but tagged within Covidence to facilitate hand searching of reference lists. Reference lists of included studies were also screened for additional studies.

### Data extraction and synthesis

Data were extracted in Excel by RMT and independently checked by a second reviewer (JPL). The data extraction template is available on GitHub.^19^ As this was a methodological systematic review interested primarily in the extent of reporting on the topic, it was relatively agnostic to the AI methods used or the study conduct. Given this fact, and the fact that the included studies were anticipated to be highly heterogenous,^3^ no formal risk of bias assessment was completed.

For our primary outcome, we followed SWiM guidance with vote counting based on direction of effect and summarising of effect estimates.^20^ We summarised effect estimates as they were reported in the majority of studies, as unstandardised relative differences (comparator/intervention); it was not possible to reliably convert the absolute difference to a standardised, comparable metric. For our secondary outcome (qualitative discussion of environmental sustainability), we summarised findings narratively. We intended to stratify findings by study characteristics of interest where possible, for example, retrospective versus prospective, study size, study subspecialty, AI task, but were unable to do so due to the small number of quantitative datapoints.

Where relevant, we documented the economic perspective taken in the evaluation (eg, healthcare provider/sector, health system, societal^21^) as well as the time horizon selected to model health and sustainability costs and benefits. Finally, we assessed which of four proposed approaches to HTA environmental impact assessment the study took: information conduit (where an HTA simply republishes existing environmental data without further assessment), integrated evaluation (where clinical, financial and environmental information is combined into a single quantitative analysis), parallel evaluation (where environmental data are analysed and presented separately from established health economic analyses) or environment-focused evaluation (where technologies which do not have a direct health benefit are evaluated solely on their environmental benefits).^14^

## Results

### Study characteristics

**Figure 1:**
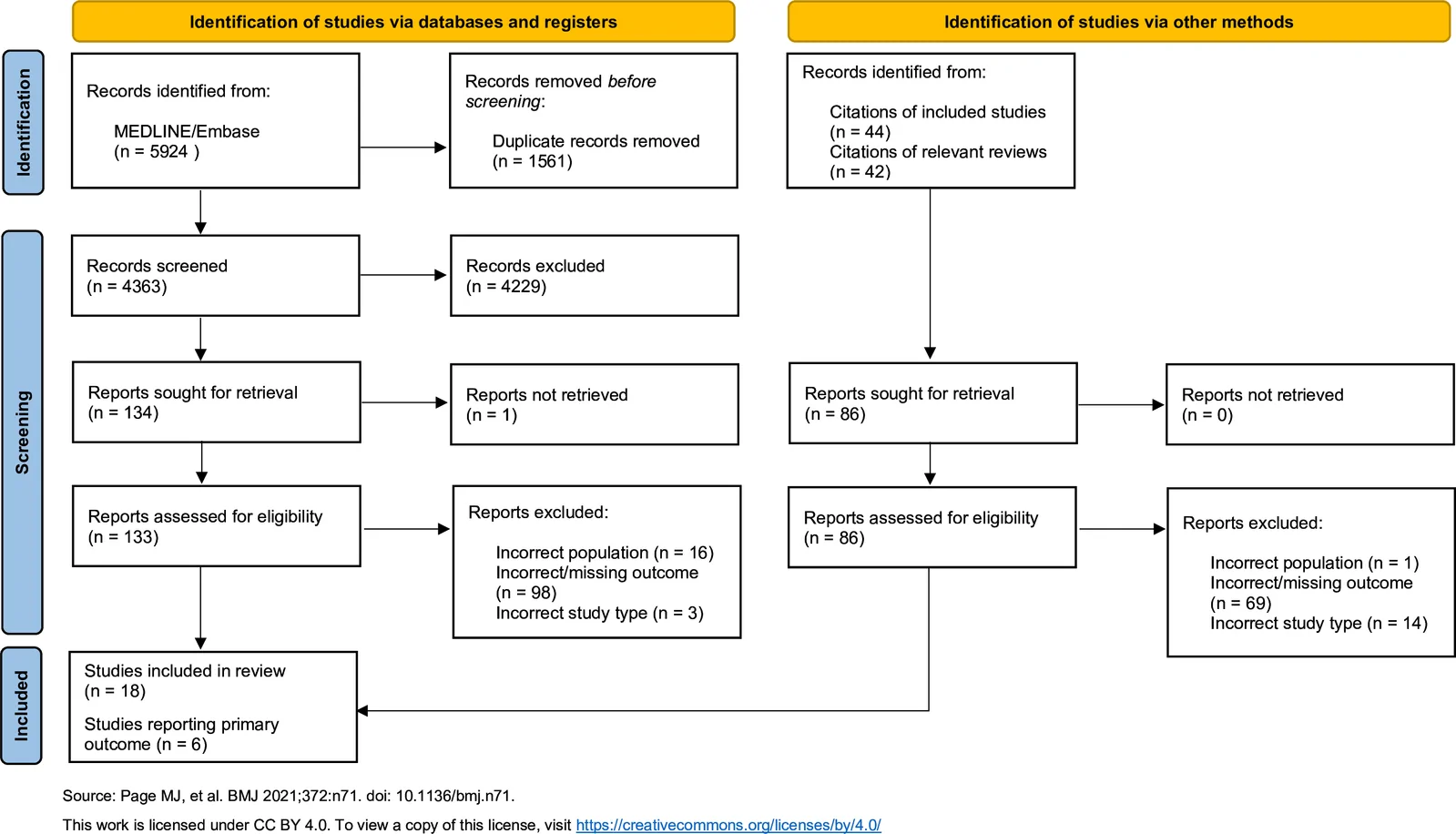
Preferred Reporting Items for Systematic Reviews and Meta-Analyses flow chart.

Of 4449 citations screened, 18 were eligible for inclusion (figure 1). Six studies^22–27^ reported our primary outcome of quantitative environmental sustainability measures (32 datapoints), and 15 studies^22 25 26 28–39^ included qualitative discussion of environmental sustainability (16 datapoints). Most of the studies were peer-reviewed journal articles (n=14, 77.78%) and the remainder were peer-reviewed conference abstracts. A list of studies excluded at full-text stage is included in online supplemental appendix C. Reviewer agreement was 97.22%, with only seven studies requiring consensus discussion at full-text stage; the kappa statistic on inter-rater reliability for full-text screening was 0.73, indicating substantial agreement.^40^

The majority of included studies (n=12, 66.67%) were from high-income countries, with only two countries contributing more than one study (the USA n=7, China n=4). Most studies analysed retrospective secondary data (n=16, 88.89%), most commonly public data (n=7, 38.89%). Interventions were most often for cancer imaging (n=8, 44.44%), and the most imaged sites were head (n=6, 33.33%), prostate (n=5, 27.78%) and chest (n=2, 11.11%). MRI was the most common imaging modality (n=13, 72.22%), with ultrasound and X-ray being the next most common (n=2, 11.1%) and one study using CT.

The most common AI task conducted as part of the intervention was classification (n=7, 38.89%), followed by segmentation (n=5, 27.78%) and prediction (n=4, 22.22%). Most studies used supervised rather than unsupervised learning (n=14, 77.78%). A range of AI architectures was used, with the most common being Green Learning (n=5, 27.78%)^41^ and various forms of U-Net (eg, two-dimensional and three-dimensional variants) (n=8, 44.44%). Full details of included studies are shown in table 1; all extracted data are available on GitHub.^19^

**Table 1:** Table of included studies

Study	AI task	Setting	Methods	Intervention	Comparator(s)	Outcome(s)	Summary of findings
**Primary outcome: quantitative measurement of environmental sustainability**							
Bohoran *et al*^22^	Aortic lumen segmentation to automate aortic distensibility calculation	UK	Retrospective analysis of data from four UK trials involving cardiac MRI (2009–2018). Adults aged 18–79 years, mix of healthy controls and those with type II diabetes, coronary artery dissection, hypertension. Sample size=376	BConvLSTM U-Net architecture used to design deliberately resource-efficient algorithm. Resource-efficient features included fewer filters in convolutional layers, fewer steps involving BConvLSTM than standard, and only one densely packed convolutional block in the final encoding step	SOTA algorithm for the same purpose, less resource efficient Unpruned algorithm for the same purpose Both trained using the same multicentre, multivendor, multidisease dataset following similar training and hyperparameter tuning procedures	CO2eq emissions (g) Energy spent (kWh) Equivalent distance travelled by car (km) All measured during final model training using Carbontracker Python tool^42^	The lightweight study algorithm outperformed the SOTA comparators on all measures of segmentation accuracy while consuming 3.9 times less fuel and generating 2.9 times less carbon emissions.
Hu *et al*^23^	Brain lesion segmentation and diagnosis prediction	China	Retrospective analysis of brain MRI data obtained from a hospital; no further information on data source or population. Sample size not reported	Hybrid Pyramid U-Net architecture and fuzzy clustering used to create more efficient algorithm for brain image processing and brain disease diagnosis prediction	Convolutional Neural Network Recurrent Neural Network Fuzzy C-Means Local Density Clustering Fuzzy C-Means Adaptive Fuzzy C-Means All included in simulations for performance comparison; no detail on how trained/tuned	Total network energy consumption (J) per simulation time/round Unclear how measured. Outcome reported only in graph, and therefore unable to be extracted for inclusion in synthesised quantitative results (authors contacted for further detail but no response)	The study algorithm had slightly reduced energy consumption when compared with five comparators applied to the same task, while achieving the highest prediction and segmentation accuracy. Authors claim this shows improved energy efficiency of fuzzy clustering as an approach, but did not explicitly link this to sustainability.
Jyoti *et al*^24^	Automatic diagnosis of COVID-19 via chest X-ray classification	India	Retrospective analysis of subset of data from public chest X-ray datasets for COVID-19 (COVIDx and COVID-CXNet), collected 2013–2017. Sample size=7468	2D tunable Q-wavelet transform based on MCA used for initial stage of image decomposition, before pretrained ResNet50 and AlexNet models used to classify images into COVID-19 and non-COVID-19. The MCA-based model is designed to process and store data more efficiently than alternatives	Use of conventional digital CMOS-based computing rather than MCA-based approach for three tasks: Image compression 128×128 Image compression 512×512 Full adder circuit	Energy consumption (nJ/pJ) Power consumption (pJ/μW) Unclear how measured. Comparator estimates appear to be sourced from existing literature rather than rerunning the models	The MCA-based approach to image decomposition resulted in up to 752 times reduction in energy consumption and up to 300 times improvement in power consumption for specific tasks compared with conventional CMOS-based computing, but the authors did not explicitly link this to sustainability.
Serrador *et al*^25^	Vertebrae segmentation	Portugal and Italy	Retrospective analysis of data from the public VerSe challenge dataset: large-scale, multidetector, multisite CT spine data collected 2013–2017. Sample size=300	Two-step segmentation. Knowledge distillation methods were implemented by training a teacher network with performance similar to that found in the literature, and this knowledge was distilled to a shallower student network	Other SOTA DL-based approaches: Several 3D U-Net Spatial-configuration Net Spine-Transformers Cyclic SS-VS-VI	CO2 emissions during segmentation of all test scans in VerSe dataset Measured using CodeCarbon package^43^	The ‘shallower’ networks used achieved comparable segmentation performance to SOTA models and demonstrated up to 89.36% reduction in carbon emissions.
Siouras *et al*^26^	ACL tear classification	Greece	Retrospective analysis of two public datasets (MRNet (2001–2012) and KneeMRI (2006–2014)) and one validation-only hospital dataset (2018). Sample size=1631	Generated highly discriminative novelty score by leveraging aleatoric semantic uncertainty as modelled in class scores outputted by a YOLOv5-nano object detection model. Second module of the pipeline is the MINIROCKET timeseries classification model to determine presence or absence of an ACL tear	Two SOTA approaches to ACL classification: Jeon *et al* 2021 Dunnhofer *et al* 2022	CO2eq emissions (g) Energy spent (kWh) Equivalent distance travelled by car (km) Authors state calculated during final training, but method of calculation not reported	The novel method outperformed classification of SOTA alternatives on accuracy and sensitivity, while generating at least 2.1 times less carbon emissions and consuming at least 2.6 times less energy.
Xiong *et al*^27^	Brain tumour segmentation	China	Retrospective analysis of data from the BraTS19 and BraTS20 public datasets, produced for multimodal brain tumour MRI segmentation challenges in 2019 and 2020. Sample size not reported	FPGA-based inference accelerator used to speed up segmentation process, quantising and retraining a 3D U-Net neural network for segmentation and merging batch normalisation layers to reduce parameter size and computational complexity	Compared with a traditional GPU-based approach: Before quantisation After quantisation	Energy consumption (W) Unclear how this was calculated, but appears to correspond to individual uses of the method, rather than the training period	FPGA-based processing provided more time efficient and accurate segmentation results, with energy efficiency 11.22 times and 82.33 times more efficient than traditional CPU and GPU-based processing. However, the authors did not explicitly link this to environmental sustainability.
**Secondary outcome: qualitative discussion of environmental sustainability**							
Abreu *et al*^28^	Prostate lesion detection	USA	Retrospective analysis of the public PI-CAI challenge dataset, an annotated multicentre dataset of bpMRIs to validate algorithm performance at detection and diagnosis of grade group ≥2 prostate lesions. Sample size=1300	Green Learning paradigm used for feature extraction, using IPHoP-II method to decompose input and feed to the classifier, which is trained to discern between prostate cancer and non-prostate cancer	None	Authors report use of Green Learning architecture, designed to be more environmentally sustainable: “We used the Green Learning paradigm for feature extraction, which offers a lightweight model size and explainable feature extraction process”	Authors note that their choice of model is lightweight compared with alternative approaches, which is linked specifically to ‘green’ aims in the selection of the Green Learning algorithm.
Bohoran *et al*^22^	Aortic lumen segmentation to automate aortic distensibility calculation	UK	As above	As above	As above	Authors explicitly link their algorithm design to sustainability repeatedly throughout the paper, for example, “The network… is resource-efficient to help promote environmentally friendly and more inclusive DL research and practices”; “By making energy usage and greenhouse gas emissions explicit, the presented work aligns with efforts to keep DL’s energy requirements and carbon cost in check”	Authors argue for energy usage and emissions to be considered key evaluation criteria in DL research, given their increasing use and significant contribution to carbon footprints: “The value of DL models should be judged by the amount of intelligence they provide per joule”.
Cacciamani *et al*^29^	Prostate lesion classification	USA	Retrospective analysis of database of men undergoing prostate MRI and biopsy from 2017 to 2023 from a separate study by the same author group (IRB# HS-13-00663). Sample size=259	Green Learning framework used for feature extraction. Data-driven automatic, unsupervised process (IPHoP-II) used to generate spatial-spectral representation of region of interest, before feature selection module is used to filter out noise and best features fed to classifier to classify according to prostate imaging reporting and data system	None	Authors report use of Green Learning architecture, designed to be more environmentally sustainable: “A novel Green Learning framework with a lightweight model and explainable feature extraction process was used”	Authors note that their choice of model is lightweight compared with alternative approaches, which is linked specifically to ‘green’ aims in the selection of the Green Learning algorithm.
Guldogan *et al*^30^	Breast lesion classification	Turkey	Retrospective analysis of data collected from women scheduled for ultrasound-guided core needle biopsy across three breast imaging centres, which were part of the same Turkish institution from August 2021 to May 2022. Sample size=530	Koios DS for Breast System used to provide probability of malignancy (BI-RADS) based on orthogonal views of lesion	None	Authors discuss implications of successful AI for sustainability in healthcare: “In conclusion, AI-based DS system performance in our study was comparable with that of breast radiologists in predicting the final assessment of breast masses. The implementation into the clinical workflow may lead to a reduction of unnecessary biopsies and short-term follow-ups, which, in turn, can contribute to sustainability in healthcare practices”	Authors link a potential reduction in unnecessary biopsies and follow-up appointments with sustainable healthcare.
Haase *et al*^31^	Prediction of full-dose GBCA images from non-contrast/low-contrast brain MRI	Germany	Prospective analysis of data from adults planned for contrast-enhanced MRI brain at single centre from August to October 2021, who received three 3D T1w scans (non-contrast, 20% standard dose, full dose). Sample size=213	Three previously published DL approaches were used to predict the full dose scan, all of which were U-Net based	U-Net algorithm performances compared with each other.	Authors discuss the environmental impacts of overuse of gadolinium-based contrast: “Reducing gadolinium-based contrast agents in brain MRI is a research topic with several motivators… [including] …lowering the impact on the environment by gadolinium-containing wastewater”	Authors state that a motivation for studies like theirs is to reduce the dose of gadolinium-containing contrast required for patients, which has patient benefits but also wider environmental benefits as gadolinium is excreted into wastewater and has harmful environmental impacts.
Kaneko *et al*^32^	Prostate lesion detection	USA	Retrospective analysis of database of men undergoing prostate MRI and biopsy from 2017 to 2023 from a separate study by the same author group (IRB# HS-13-00663). Sample size=259	Green Learning framework used for feature extraction and ROI classification, specifically the IPHoP-II process. Independent modules used for peripheral and transition zones	None	Authors report use of Green Learning architecture, designed to be more environmentally sustainable: “For feature extraction and ROI classification, we adopt the Green Learning paradigm, and specifically the IPHoP-II feature extraction process”	Authors use the Green Learning model, which is designed to be lightweight and linked explicitly to green aims through its name. However, authors themselves do not comment on model complexity or sustainability.
Kaneko *et al*^33^	Automated prostate segmentation	USA	Retrospective analysis of database of men undergoing prostate MRI and biopsy from 2017 to 2023 from a separate study by the same author group (IRB# HS-13-00663). Sample size=119	Novel two-stage automatic Green Learning-based model. First stage segments prostate gland and second stage zooms in to delineate the transition zone and peripheral zone. Both stages share a lightweight feed-forward encoder-decoder Green Learning system	None	Authors report use of Green Learning architecture, designed to be more environmentally sustainable: “A lightweight feed-forward encoder-decoder model based on Green Learning can precisely segment the whole prostate”	Authors note that their choice of model is lightweight compared with alternative approaches, which is linked specifically to ‘green’ aims in the selection of the Green Learning algorithm.
Lee *et al*^34^	Ultrasound image reconstruction and inference	South Korea	Prospective analysis of data from volunteers, who received abdominal ultrasound; no information provided on recruitment. Most images normal. Sample size=25	DL edge-computing method for mobile device-based ultrasound scanners that perform image reconstruction and inference for object classification. Four architectures used: AlexNet, ShallowNet, MobileNet and Xception	All four algorithms trained on RSC-generated dataset versus standard ultrasound dataset	Authors discuss that the rollout of cloud-based AI is limited by a number of factors including energy consumption: “in a cloud-based ultrasonic artificial intelligence system, the use of POCUS is limited due to information security, network integrity and significant energy consumption”	Authors note that cloud-based systems are associated with significant energy consumption, which is particularly problematic in resource-poor settings. They propose their algorithms as an alternative that can run without requiring internet access.
Li *et al*^35^	COVID-19 detection on chest X-ray	China	Retrospective analysis of data from five public data repositories of COVID-19 images, creating the COVIDx dataset. Sample size=16 689	FedFocus, a FL platform which allows clients to collaboratively train models without exposing their source data. ResNet18 architecture used for training model in each client	Central training (not possible in real world due to data privacy restrictions) Local training (client takes training set allocated to it for individual training) FedAvg (leading method in FL to minimise training loss)	Authors discuss reducing energy consumption as a goal throughout, for example, “Current FL-based COVID-19 detection methods tend to deploy the most popular methods FedAvg or FedSGD, and optimize multiobjectives including delay, energy consumption, and privacy”; “Compared with other deeper residual network models, ResNet18 has fewer parameters effectively relieve the pressure of network bandwidth and energy consumption”	Authors selected an AI architecture that is more lightweight with fewer parameters and explicitly tie this to potential reduction in energy consumption of their final model.
Magoulianitis *et al*^36^	Prostate cancer segmentation	USA	Retrospective analysis of public dataset of bpMRI data generated for the PI-CAI prostate segmentation challenge. Sample size=1000	Two-stage method which first generates voxel-wise predictions to deduce prostate cancer probability of each voxel, and second derives an anomaly score heatmap providing initial indication of suspicious looking areas with high probability of cancer	Other SOTA DL approaches: SwinTransformer nnDetection nnU-Net U-Net	Authors report use of Green Learning architecture, designed to be more environmentally sustainable: “It [the approach] adopts the recently introduced Green Learning paradigm, which offers a small model size and low complexity”; “this stresses one of the Green Learning framework key benefits which is to offer lightweight and environment-friendly solutions”	Authors note that their choice of model, named ‘Green Learning’ to emphasise its reduced environmental impact, is lightweight compared with alternative approaches using DL, and they explicitly link this design choice to environmental benefits.
Pasumarthi *et al*^37^	Prediction of full-dose GBCA images from non-contrast/low-contrast brain MRI	USA	Retrospective analysis of data from three sites of patients undergoing specific enhanced scanning protocol on brain MRI. Sample size=640	U-Net based model to predict contrast-enhanced images from corresponding precontrast and low-contrast images. Quantitative tumour segmentation performed on cases with abnormal enhancements	Low-dose MRI Full-dose MRI (compared segmentation outcome)	Authors discuss the environmental impacts of overuse of GBCA: “Environmental sustainability concerns are also being raised as gadolinium is an emerging water pollutant”	Authors link a reduction in use of GBCA with a reduction in water pollution.
Serrador *et al*^25^	Vertebrae segmentation	Portugal and Italy	As above	As above	As above	Authors highlight the large environmental impact of existing models and note the favourability of their approach, for example, “The size and complexity of these DL models contribute to a heightened environmental footprint”; “[our method] … contribut(ed) to reduced environmental impact as outlined in the European Commission’s Ethics Guidelines for Trustworthy AI”	Authors aimed to create models that were explicitly more lightweight and had smaller environmental footprints than comparative algorithms, linking this goal to ethical guidance on building trustworthy AI.
Siouras *et al*^26^	ACL tear classification	Greece	As above	As above	As above	Both environmental sustainability and energy usage were explicitly highlighted throughout the article, for example, “concerns over the environmental footprint of DL research and its impact on accelerating global climate change have been growing”; “[this] research paves the way for future advancements in the DL-powered MRI-based injury detection, emphasizing environmental sustainability alongside technological innovation”; “previous papers disregarded the compute demand by the DL model. Yet, this is a significant matter since these computations necessitate mind-boggling amounts of generated power for fuelling them”	Authors selected a model structure that is less computationally intensive and specifically link this to environmental sustainability, highlighting wider discussion within DL literature on green/climate goals. The authors also highlight the current lack of consideration of computational demands of DL models and express the view that this is an oversight given the high energy consumption associated with complex models.
Wang *et al*^38^	Prediction of full-dose GBCA images from non-contrast brain MRI	China	Retrospective analysis of data from patients undergoing brain MRI at two hospitals in China from 2019 to 2021. Unclear if recruited specifically for this study. Sample size=221	3D U-Net-like generator producing 3D isotropic full-contrast T2Flair images from 2D anisotropic non-contrast T2Flair image stacks	3D Res U-Net Bayes U-Net Convnets	Authors discuss the environmental impacts of overuse of GBCA: “In addition, gadolinium has been reported as an emerging water pollutant and may harm the environment”	Authors link a reduction in use of GBCA with a reduction in water pollution.
Xie *et al*^39^	Prediction of full-dose GBCA images from non-contrast brain MRI	USA	Retrospective analysis of the public BraTS20 dataset, produced for multimodal brain tumour segmentation challenge in 2020. Sample size=369	Two sequential networks: A retina U-Net trained to derive semantic features from non-contrast MR images to represent tumour regions A synthesis module trained to generate synthetic contrast-enhanced MR images from semantic feature maps and non-contrast images	None	Authors discuss the environmental impacts of overuse of GBCA: “Although GBCAs are generally thought to be safe, various health and environmental concerns have been raised recently about their use in MR imaging”; “Debates on toxicity of gadolinium deposition in the brain, breast, and body, as well as environmental issues, have been raised by most recent studies”	Authors designed a machine learning approach that would reduce the amount of GBCA required in MRI studies, and link the desire to do so with environmental as well as health concerns about contrast use.

ACL, anterior cruciate ligament; AI, artificial intelligence; BI-RADS, Breast Imaging Reporting and Data System; CMOS, complementary metal oxide semiconductor circuits; CO2eq, carbon dioxide equivalent; CPU, central processing unit; 2D, two-dimensional; 3D, three-dimensional; DL, deep learning; DS, decision support; FL, Federated Learning; FPGA, field programmable gate array; GBCA, gadolinium-based contrast agent; GPU, graphics processing unit; IPHoP-II, integrated Phage-Host Prediction tool; MCA, memristive crossbar array; MR, magnetic resonance; PI-CAI, prostate imaging-cancer artificial intelligence; POCUS, point-of-care ultrasound; ROI, region of interest; RSC, reverse scan conversion; SOTA, State-of-the-art; T1w, T1-weighted.

### Primary outcome: quantitative measures of sustainability

The six studies which quantitatively measured environmental sustainability reported on carbon emissions (CO2eq, n=3, 16 datapoints) or other measures of energy consumption (eg, joules/watts, n=5, 16 datapoints), and all reported on algorithms *a priori* intended to be more efficient or less computationally intensive than competitors (table 1). Two studies reported use of carbon tracking software packages^42 43^ to generate their outcomes; no others reported how emissions or energy consumption were calculated. One study reporting on energy consumption^23^ could not be included in quantitative analysis, as findings were only reported in a graph; we attempted to contact the authors for the underlying data, but this was not successful.

We identified no studies reporting on any other sustainability measures of interest, for example, water/mineral usage, waste generation/disposal or impacts on material environments. The comparators in all six studies were other AI interventions applied to the same clinical task, with no studies comparing sustainability outcomes for an AI-enabled pathway versus standard-of-care. All quantitative studies took a healthcare provider perspective and conducted a parallel evaluation of environmental outcomes (ie, analysing and presenting environmental data alongside health analyses, rather than directly integrating this into health economic models or having an entirely separate environment-focused evaluation^14^). Half of the studies measured outcomes during algorithm training only, with the other half measuring outcomes during deployment only.

For all primary outcome datapoints, the AI intervention of interest had a beneficial effect direction (ie, was shown to produce fewer carbon emissions and consume less energy than the comparator models) when applied to the same clinical tasks, while maintaining similar or improved clinical performance (table 1). Intervention algorithms generated from 2.19 to 17.15 times less carbon emissions (median 7.81) and from 1.60 to 751.62 times less energy consumption (median 3.22) (table 2). The heterogeneity of included measures precluded further quantitative synthesis, for example, meta-analysis or standardisation.

**Table 2:** Summary of relative differences (ratio)

Outcome	Mean	Median	Minimum	Maximum	IQR	Count (n)
Carbon emissions	8.39	7.81	2.19	17.15	7.07	16
Energy use	69.82	3.22	1.60	751.62	5.56	16

### Secondary outcome: qualitative discussion of sustainability

The 15 studies that included qualitative discussion of sustainability discussed energy usage (n=8), environmental sustainability or climate change (n=4), and impacts on material environment (n=4). Studies which mentioned material environment impacts all focused on the use of gadolinium-based contrast agent (GBCA) and referred to its role as a wastewater pollutant. In most papers, particularly those that did not include quantitative measurements of sustainability, discussion of sustainability was very limited: 75% of these (n=9) included only a single sentence on sustainability, either mentioning the use of a lightweight algorithm designed to be ‘green’, or briefly noting environmental concerns related to energy consumption or pollutants. The extracted data are included in full in table 1.

The three studies that reported both our primary and secondary outcome^22 25 26^ included much more in-depth discussion of the environmental sustainability aspects of AI in radiology, with this being a clear focus of the paper. These studies discussed their own ‘green’ intentions with their models and referenced the wider clinical and policy context, making recommendations for both greater computational efficiency of algorithms to reduce the carbon footprint of AI in radiology, and increased reporting of sustainability outcomes as standard alongside other common outcomes. Interestingly, however, the three other studies that reported quantitative measures (all measures of energy consumption) did not explicitly link this to environmental sustainability at any point in their papers.^23 24 27^

## Discussion

Across a 9-year period when AI-related publications in clinical radiology were rapidly increasing from ~100 to almost 4000/year,^44^ our methodological systematic review found very few studies of AI interventions in radiology discussing environmental sustainability (n=15) and even fewer (n=6) quantitatively measuring sustainability outcomes. Quantitative outcomes reported included carbon emissions and energy consumption (eg, kilowatt hours or joules), with studies finding that their algorithms were able to provide similar or better clinical performance while on average emitting eight times less carbon and consuming three times less energy. Half of these studies did not explicitly link their measurement of such outcomes to environmental sustainability, and all studies were of algorithms that were specifically designed to be lightweight and less computationally intensive than competitors. Other than in the three papers which had a deliberate focus on environmental sustainability,^22 25 26^ qualitative discussion of the topic was very superficial, with most studies including only a single sentence on the topic. Interestingly, a common topic raised in four papers was the potential for AI technologies to reduce the amount of GBCA required for scans, with this having the environmental co-benefit of reducing gadolinium pollution of wastewater.^31 37–39^

As noted in our introduction, it has been suggested that environmental assessment is poorly integrated into HTAs.^13 14^ Our findings suggest that this is certainly the case for HTAs focusing specifically on evaluating AI technologies, at least within clinical radiology. In addition, despite the availability of existing frameworks—such as life cycle assessment^45^ or integrated assessment^14^—the small number of studies reporting quantitative outcomes showed little consistency in what was reported. In several studies, a lack of reporting made it challenging to identify how or when these outcomes had been measured (table 1). This may suggest a requirement for effective guidance and frameworks that are more tailored to AI technology evaluations in healthcare.^46^ The most comprehensive studies^22 25^ leveraged software tools such as Carbontracker or CodeCarbon,^42 43^ which allowed them to report more standardised outcomes, for example, CO2eq emissions, or energy spent in kWh. More widespread awareness and adoption of these tools, which are designed to be simple to integrate into existing software pipelines and scalable, could also be beneficial in standardising outputs of environmental assessments within HTAs of AI healthcare technology.^26^ However, this would require upskilling of the clinical and technical workforce to ensure that integrating such software is seen as both feasible and valuable.

Of interest, many studies of algorithms designed for a similar purpose as those in our included studies (ie, to be lightweight) were excluded at full-text stage because the study authors did not link this intention to sustainability; instead, this was linked to goals such as promotion of mobile use of AI,^47^ or improved use in resource-constrained settings.^48^ This suggests that while there is clearly cost pressure to produce higher efficiency algorithms with lower computational demand, and while movement in this direction also has benefits for environmental sustainability, a desire for environmental sustainability as defined in this review is not currently a key driver of this. However, it demonstrates that if improved computational efficiency is seen as providing a competitive edge, then developers will focus on achieving this; perhaps a sign that, if incentives were to be altered in relation to sustainability, there could be a shift in the focus of developers and deployers.^22 49^

A shift in incentives is potentially within the influence of decision-makers, as well as regulators, clinical guideline developers and research funders, many of whom have outlined in their own strategies a clear commitment to incorporating environmental sustainability assessment into their decision-making processes in future.^50 51^ Radiology as a specialty has also expressed a growing interest in AI’s carbon footprint in recent years.^5 15 16^ Clear, structured guidance on how to report these outcomes, alongside a much firmer expectation from these bodies that they *should* be reported, could ideally lead to an increase in clinical radiology AI studies that transparently measure and report on the environmental performance of algorithms, allowing them to be more comprehensively judged according to ‘the amount of intelligence they provide per joule’.^22^

As the first on this topic, we believe our study has some important strengths. We closely followed a preregistered systematic review protocol and adhered to gold standard Cochrane guidance in our conduct, analysis and presentation of results.^17 18 20^ We built on an existing tested search strategy^3^ and believe we are the first to try to quantify the extent to which environmental sustainability is being measured in AI radiology literature to date. However, there are some important limitations to acknowledge. First, modifying the search strategy to find studies that considered sustainability was challenging. We were required to add terms for sustainability to keep the search outputs manageable, and while we allowed for these terms to be included anywhere that was indexed, it is possible we may have missed some studies that briefly mention sustainability. However, as we note above, studies falling into this category did not contribute much to our synthesis, and we also performed citation searching to increase our yield, identifying two additional studies (figure 1). Second, as noted in the methods we have not formally measured study quality, so could not comment on how this may have influenced findings. However, given the consistency of the quantitative findings and small number of datapoints, it is unlikely this would have changed our conclusions. Finally, our ability to standardise effect sizes was limited by the uncommon and inconsistent measures used within the quantitative studies, meaning that we report only relative differences of the included measures.

Our findings have implications for both research and policy (table 3). We have identified a clear research gap for studies that compare AI-enabled clinical pathways to standard-of-care or care as usual, rather than just state-of-the-art alternative AI approaches; without this, it is impossible to adequately judge the potential environmental costs and benefits brought by the AI technologies. In terms of further evidence synthesis, given the pace of development (with a majority of our papers publishing from 2023 onwards), a living systematic review could be a useful methodology to explore this topic further in future,^52^ particularly with the recent rapid increase in papers using generative AI including large language models.^53^ In addition, it would be of interest to apply our methods to other fields where AI technologies are beginning to be used more widely but where there is perhaps more awareness of environmental sustainability as a topic, for example, public health, to see whether this influences findings.^54^ From a policy perspective, we have identified a clear need for a standardised methodology and reporting framework for environmental outcome assessment specific to AI, as evaluations are sparse and inconsistent in their methods and reporting. It is clear that reporting of these outcomes is not happening adequately at present, and it may be that policymakers must incentivise this in some way, for example, by ensuring there is some competitive advantage to reporting carbon emissions and energy consumptions of models when developers seek regulatory approval.^22^ In addition, given that many governments and healthcare systems have specific policies in place to mitigate against climate change, they risk directly undermining their efforts to do so if they pursue widespread AI deployment without adequate mitigation of environmental impact.

**Table 3:** Summary of key recommendations

Recommendation	Target group(s)
Computational complexity and energy efficiency of AI algorithms should be more explicitly taken into account when procuring, training and testing these technologies in clinical settings	Researchers; clinicians; healthcare managers
Evaluations of AI deployment in clinical radiology must better integrate measurement and reporting of environmental sustainability, particularly when comparing AI-augmented pathways to standard-of-care	Researchers; clinicians
Development of effective guidance and/or frameworks for environmental evaluation of AI technology in healthcare would be beneficial, and should include recommendations on preferred metrics for reporting to improve comparability across studies	Regulators; clinical guideline developers; researchers; clinicians
AI developers seeking regulatory approval or access to clinical data for testing/deployment should be better incentivised to consider, measure and report environmental sustainability outcomes	Regulators; clinical guideline developers; funding bodies; clinicians
To facilitate increased reporting and ensure outcomes are more comparable across studies, there should be more widespread awareness and adoption of software tools designed to measure environmental outcomes of algorithms, for example, Carbontracker, CodeCarbon^42 43^	AI developers; researchers; clinicians

AI, artificial intelligence.

In conclusion, despite rising concerns about the potential impacts of AI technologies on the environment, measurement of sustainability is poorly integrated into current evaluations of AI in clinical radiology, except where reduced computational demand was an explicit selling point of the algorithm. No studies compared AI with standard-of-care, making it impossible to ascertain whether AI-augmented pathways (particularly those focused on prevention) have potential to reduce future carbon costs associated with healthcare utilisation. Uncritically continuing the widespread rollout of AI in healthcare without evaluating its potential environmental impacts risks worsening the climate crisis, with major implications for global health. Researchers and clinicians should aim, where possible, to quantify the environmental impacts of the technologies they are evaluating, establishing the costs and benefits for the climate as well as for health. To meet their stated aims on sustainability, funders and governance bodies should consider how to promote better integration of environmental impact assessment into AI health research and evaluation.

10.1136/bmjdhai-2025-000073.supp2 Supplementary data

## Data Availability

Data are available in a public, open access repository. The study protocol is published on PROSPERO: https://www.crd.york.ac.uk/PROSPERO/view/CRD42024601818. Data extracted from included studies (including data used for all analyses) and analytic code are available on GitHub: https://github.com/rachelmthomson/thomson-AI-sustainability-sr.
