# Supplementary material for: Evaluating the environmental sustainability of AI in radiology: a systematic review of current practice
Source: BMJ Digit Health Ai. 2025 Oct 3;1(1):e000073. doi: 10.1136/bmjdhai-2025-000073 (PMC13492543; doi:10.1136/bmjdhai-2025-000073)
Supplement: Supplementary file 1 [file bmjdhai-1-1-bmjdhai-2025-000073-s001.pdf]

Appendix A: Protocol deviations

The only deviations from the protocol as published were:

1. No automated/AI tools were required in the searching of full texts
2. The sparsity of extracted quantitative data meant meta-analysis could not be conducted as planned

Appendix B: Search strategy

Databases: Ovid MEDLINE(R) ALL 1946 to November 04, 2024; Embase 1947-Present, updated daily

1	(Artificial intelligence or Machine learning or Support vector machine or SVM or CNN or RNN or LSTM or ResNet or DenseNet or Unet or U-net or DNN or Neural network* or Convolutional network* or Deep learn* or Semantic segmentation or Ensemble or Classification tree or regression tree or probability tree or nearest neighbo* or fuzzy logi* or random forest or kernel or k-means or naive bayes).ti,ab.	814056
2	limit 1 to english language	801371
3	limit 2 to human	434348
4	limit 3 to yr="2015 -Current"	377785
5	(X-ray or X-ray* or Radiography or Radiograph* or Computed tomography or CT or CAT or CTA or Computerized axial tomography or Magnetic resonance imag* or MRI or MR or Magnetic resonance angio* or MRA or Scintigraphy or DMSA or Ultrasound* or Sonograph* or PET or Positron Emission Tomography or SPECT or Single-photon emission or Single photon emission or mammogra*).ti,ab.	5701203
6	limit 5 to english language	5186930
7	limit 6 to human	3614029
8	limit 7 to yr="2015 -Current"	1885467
9	4 and 8	79395
10	(Sustainab* or green* or environment* or climate or global warming or global heating or ecolog* or net zero or carbon* or emissions or life cycle assessment or LCA or energy or water or air or mineral or waste).mp. [mp=ti, bt, ab, ot, nm, hw, fx, kf, ox, px, rx, ui, sy, ux, mx, tn, dm, mf, dv, dq]	11085266
11	limit 10 to english language	10412218
12	limit 11 to human	3979734

13	limit 12 to yr="2015 -Current"	2026674
14	9 and 13	5924

Appendix C: List of studies excluded at full text stage

**Reference**	**Reason**
AHMED, R., MADDIKUNTA, P. K. R., GADEKALLU, T. R., ALSHAMMARI, N. K. & HENDAOUI, F. A. 2024. Efficient differential privacy enabled federated learning model for detecting COVID-19 disease using chest X-ray images. *Frontiers in Medicine,* 11.	Incorrect/missing outcome
ALSOLAMY, M., NADEEM, F., AZHARI, A. A., ALSOLAMI, W. & AHMED, W. M. 2024. Automated detection and labeling of posterior teeth in dental bitewing X-rays using deep learning. *Computers in Biology and Medicine,* 183.	Incorrect/missing outcome
AMIN, M. N., KAMAL, R., FAROUK, A., GOMAA, M., RUSHDI, M. A. & MAHMOUD, A. M. 2023. An efficient hybrid computer-aided breast cancer diagnosis system with wavelet packet transform and synthetically-generated contrast-enhanced spectral mammography images. *Biomedical Signal Processing and Control,* 85.	Incorrect/missing outcome
ANITHA, T. & KALAISELVI, T. 2022. MRI Brain Tumor Classification Using Computational Intelligence Technique. *NeuroQuantology,* 20(14)**,** 1430-1438.	Incorrect/missing outcome
BHATT, H. & SHAH, M. 2023. A Convolutional Neural Network ensemble model for Pneumonia Detection using chest X-ray images. *Healthcare Analytics,* 3.	Incorrect/missing outcome
BYRA, M., GALPERIN, M., OJEDA-FOURNIER, H., OLSON, L., O'BOYLE, M., COMSTOCK, C. & ANDRE, M. 2019. Breast mass classification in sonography with transfer learning using a deep convolutional neural network and color conversion. *Medical Physics,* 46**,** 746-755.	Incorrect/missing outcome
CAO, D., ZHANG, R. & ZHANG, Y. 2024. MFLUnet: multi-scale fusion lightweight Unet for medical image segmentation. *Biomedical Optics Express,* 15(10)**,** 5574-5591.	Incorrect/missing outcome
CASE, J., JOHN, F., MCGHIE, A., SOMAN, P., COURTER, S., CASE, K., SCHWARTZ, R., BERMAN, D. & BATEMAN, T. 2022. Validation of a Source-Less, Deep Learning Technique for Attenuation Correction of Stressonly CZT-SPECT studies. *Journal of Nuclear Medicine. Conference: Society of Nuclear Medicine and Molecular Imaging Annual Meeting, SNMMI,* 63.	Incorrect population
CELAYA, A., ACTOR, J., MUTHUSIVARAJAN, R., GATES, E., CHUNG, C., SCHELLINGERHOUT, D., RIVIERE, B. & FUENTES, D. 2021. Small convolutional neural networks for efficient 3D medical image segmentation. *Medical Physics,* 48(6)**,** e409-e410.	Incorrect/missing outcome
CROSBY, J., RHINES, T., LI, F., MACMAHON, H. & GIGER, M. 2020. Deep convolutional neural networks in the classification of dual-energy thoracic radiographic views for efficient workflow: Analysis on over 6500 clinical radiographs. *Journal of Medical Imaging,* 7(1) .	Incorrect/missing outcome
DANG, T., FERMIN, A. S. R. & MACHIZAWA, M. G. 2022. oFVSD: A Python package of optimized forward variable selection decoder for high-dimensional neuroimaging data. *bioRxiv.,* 25.	Incorrect population
DANG, T., FERMIN, A. S. R. & MACHIZAWA, M. G. 2023. oFVSD: a Python package of optimized forward variable selection decoder for high-dimensional neuroimaging data. *Frontiers in Neuroinformatics,* 17.	Incorrect/missing outcome
DAS, V., DANDAPAT, S. & BORA, P. K. 2019. A novel diagnostic information based framework for super-resolution of retinal fundus images. *Computerized Medical Imaging & Graphics,* 72**,** 22-33.	Incorrect/missing outcome

DEL GAIZO, A. J., OSBORNE, T. F., SHAHOUMIAN, T. & SHERRIER, R. 2024. Deep Learning to Detect Intracranial Hemorrhage in a National Teleradiology Program and the Impact on Interpretation Time. *Radiology Artificial intelligence,* 6, e240067.	Incorrect/missing outcome
DIAS, B. S. S., QUERRER, R., FIGUEIREDO, P. T., LEITE, A. F., DE MELO, N. S., COSTA, L. R., CAETANO, M. F. & FARIAS, M. C. Q. 2025. Osteoporosis screening: Leveraging EfficientNet with complete and cropped facial panoramic radiography imaging. *Biomedical Signal Processing and Control,* Part B. 100.	Incorrect/missing outcome
DOU, Q., YU, L., CHEN, H., JIN, Y., YANG, X., QIN, J. & HENG, P. A. 2017. 3D deeply supervised network for automated segmentation of volumetric medical images. *Medical Image Analysis,* 41, 40-54.	Incorrect/missing outcome
DU, J., WANG, J., GAI, X., SUI, Y., LIU, K. & YANG, D. 2023. Application of intelligent X-ray image analysis in risk assessment of osteoporotic fracture of femoral neck in the elderly. *Mathematical Biosciences & Engineering: MBE,* 20, 879-893.	Incorrect/missing outcome
EKMAN, A., JENSEN, C. F. S. & ELENKOV, A. 2024. Identifying Seminiferous Tubules for Sperm Retrieval during Microdissection Testicular Sperm Ectraction (M-Tese) Using Deep Learning. *Fertility and Sterility,* 122(4 Supplement), e268.	Incorrect/missing outcome
EMAMI, H., DONG, M., NEJAD-DAVARANI, S. P. & GLIDE-HURST, C. K. 2018. Generating synthetic CTs from magnetic resonance images using generative adversarial networks. *Medical Physics,* 45(8), 3627-3636.	Incorrect population
FALLAH, F., WALTER, S. S., BAMBERG, F. & YANG, B. 2019. Simultaneous Volumetric Segmentation of Vertebral Bodies and Intervertebral Discs on Fat-Water MR Images. *IEEE Journal of Biomedical & Health Informatics,* 23, 1692-1701.	Incorrect/missing outcome
FAN, S., WU, E., CAO, M., XU, T., LIU, T., YANG, L., SU, J. & LIU, J. 2023. Flexible In-Ga-Zn-N-O synaptic transistors for ultralow-power neuromorphic computing and EEG-based brain-computer interfaces. *Materials Horizons,* 10, 4317-4328.	Incorrect population
FANG, L. & WANG, X. 2023. Multi-input Unet model based on the integrated block and the aggregation connection for MRI brain tumor segmentation. *Biomedical Signal Processing and Control,* Part 1. 79.	Incorrect/missing outcome
FENNETEAU, A., BOURDON, P., HELBERT, D., FERNANDEZ-MALOIGNE, C., HABAS, C. & GUILLEVIN, R. 2021. Investigating efficient CNN architecture for multiple sclerosis lesion segmentation. *Journal of Medical Imaging,* 8(1) .	Incorrect/missing outcome
FISCHER, M., BARTLER, A. & YANG, B. 2024. Prompt tuning for parameter-efficient medical image segmentation. *Medical Image Analysis,* 91, 103024.	Incorrect/missing outcome
GLANG, F., DESHMANE, A., PROKUDIN, S., MARTIN, F., HERZ, K., LINDIG, T., BENDER, B., SCHEFFLER, K. & ZAISS, M. 2020. DeepCEST 3T: Robust MRI parameter determination and uncertainty quantification with neural networks-application to CEST imaging of the human brain at 3T. *Magnetic Resonance in Medicine,* 84, 450-466.	Incorrect population
GONG, Z., KHATTAR, N., KIELY, M., TRIEBSWETTER, C. & BOUHRARA, M. 2023. Rapid whole-brain high-resolution myelin water fraction mapping from extremely under-sampled magnetic resonance imaging data using deep neural network. *medRxiv.,* 09.	Incorrect study type
HANUSSEK, M., BARTUSCH, F. & KRUGER, J. 2021. Performance and scaling behavior of bioinformatic applications in virtualization environments to create awareness for the efficient use of compute resources. *PLoS Computational Biology,* 17, e1009244.	Incorrect population
HARDY, N. P., EPPERLEIN, J. P., DALLI, J., SARKAR, A., ZHUK, S., MACAONGHUSA, P., KHAN, M. F., BRANNIGAN, A., MULSOW, J., NEARY, P. M. & CAHILL, R. A. 2022. Classification and delineation of	Incorrect/missing outcome

rectal tumours through artificial intelligence metabolic characterisation. *Surgical Endoscopy,* 36(1 SUPPL), S5.	
HEINRICH, M. P., BLENDOWSKI, M. & OKTAY, O. 2018. TernaryNet: faster deep model inference without GPUs for medical 3D segmentation using sparse and binary convolutions. *International Journal of Computer Assisted Radiology & Surgery,* 13, 1311-1320.	Incorrect/missing outcome
HENSCHEL, L., CONJETI, S., ESTRADA, S., DIERS, K., FISCHL, B. & REUTER, M. 2020. FastSurfer - A fast and accurate deep learning based neuroimaging pipeline. *Neuroimage,* 219, 117012.	Incorrect/missing outcome
HEO, H. Y., SINGH, M., YEDAVALLI, V., JIANG, S. & ZHOU, J. 2024. CEST and nuclear Overhauser enhancement imaging with deep learning-extrapolated semisolid magnetization transfer reference: Scan-rescan reproducibility and reliability studies. *Magnetic Resonance in Medicine,* 91, 1002-1015.	Incorrect/missing outcome
HIGASHIGAWA, T., ICHIKAWA, Y., NAKAJIMA, K., KOBAYASHI, T., DOMAE, K., YAMAZAKI, A., KATO, N., OUCHI, T., KATO, H. & SAKUMA, H. 2024. Low energy virtual monochromatic CT with deep learning image reconstruction to improve delineation of endoleaks. *Clinical Radiology,* 79, e1260-e1267.	Incorrect population
HOIDN, O., MISHRA, A. A. & MEHTA, A. 2023. Physics constrained unsupervised deep learning for rapid, high resolution scanning coherent diffraction reconstruction. *Scientific Reports,* 13(1), 22789.	Incorrect population
HU, P., WU, F., PENG, J., BAO, Y., CHEN, F. & KONG, D. 2017. Automatic abdominal multi-organ segmentation using deep convolutional neural network and time-implicit level sets. *International Journal of Computer Assisted Radiology & Surgery,* 12, 399-411.	Incorrect/missing outcome
IBRAHIM, A. R. & BROUMANDNIA, A. 2024. Developing Deep Learning Algorithms for Medical Image Processing and Analysis. *African Journal of Biological Sciences (South Africa),* 6(15), 1118-1142.	Incorrect/missing outcome
ISHIDA, J., SAITO, A. & SHIMIZU, A. 2017. Multi-modality statistical model between a thoracic CT volume and photographs of lung cryosections of a cadaver. *International Journal of Computer Assisted Radiology and Surgery,* 12(1 Supplement 1), S192-S193.	Incorrect population
JUNEJA, S., JUNEJA, A., DHIMAN, G., BEHL, S. & KAUTISH, S. 2021. An Approach for Thoracic Syndrome Classification with Convolutional Neural Networks. *Computational & Mathematical Methods in Medicine,* 2021, 3900254.	Incorrect/missing outcome
JUNG, S. K., LIM, H. K., LEE, S., CHO, Y. & SONG, I. S. 2021. Deep active learning for automatic segmentation of maxillary sinus lesions using a convolutional neural network. *Diagnostics,* 11(4) .	Incorrect/missing outcome
KAHALI, S., KOTHAPALLI, S., XU, X., KAMILOV, U. S. & YABLONSKIY, D. A. 2023. Deep learning-based Accelerated and Noise-Suppressed Estimation (DANSE) of quantitative Gradient-Recalled Echo (qGRE) magnetic resonance imaging metrics associated with human brain neuronal structure and hemodynamic properties. *NMR in Biomedicine,* 36, e4883.	Incorrect population
KANEKO, M., MAGOULIANITIS, V., RAMACCIOTTI, L. S., RAMAN, A., PARALKAR, D., CHEN, A., CHU, T. N., YANG, Y., XUE, J., YANG, J., LIU, J., JADVAR, D. S., GILL, K., CACCIAMANI, G. E., NIKIAS, C. L., DUDDALWAR, V., JAY KUO, C. C., GILL, I. S. & ABREU, A. L. 2024. The Novel Green Learning Artificial Intelligence for Prostate Cancer Imaging: A Balanced Alternative to Deep Learning and Radiomics. *Urologic Clinics of North America,* 51, 1-13.	Incorrect study type
KANG, S. K., SHIN, S. A., SEO, S., BYUN, M. S., LEE, D. Y., KIM, Y. K., LEE, D. S. & LEE, J. S. 2021. Deep learning-Based 3D inpainting of brain MR images. *Scientific Reports,* 11, 1673.	Incorrect population
KATORN, C., ISHIYAMA, K., MAGOTAS, K. & HIRATA, K. 2021. Strategy to estimate the time-activity curve of arterialplasma in 18F-FDG dynamic brain PET study with Convolutional Neural Network.	Incorrect/missing outcome

Journal of Nuclear Medicine. Conference: Society of Nuclear Medicine and Molecular Imaging Annual Meeting, SNMMI, 62.	
KHAN, U., MENTO, F., NICOLUSSI GIACOMAZ, L., TREVISAN, R., SMARGIASSI, A., INCHINGOLO, R., PERRONE, T. & DEMI, L. 2022. Deep Learning-Based Classification of Reduced Lung Ultrasound Data From COVID-19 Patients. *IEEE Transactions on Ultrasonics Ferroelectrics & Frequency Control,* 69**,** 1661-1669.	Incorrect/missing outcome
KHOSRAVI, M., ESMAEILI, M., MOGHADDAM, Y. J., KESHTKAR, A., JALILI, J. & NASRABADI, H. T. 2022. A Robust Machine learning based method to classify normal and abnormal CT scan images of mastoid air cells. *Health and Technology,* 12(2)**,** 491-498.	Incorrect/missing outcome
KICKINGEREDER, P., ISENSEE, F., TURSUNOVA, I., PETERSEN, J., NEUBERGER, U., BONEKAMP, D., BRUGNARA, G., SCHELL, M., KESSLER, T., FOLTYN, M., HARTING, I., SAHM, F., PRAGER, M., NOWOSIELSKI, M., WICK, A., NOLDEN, M., RADBRUCH, A., DEBUS, J., SCHLEMMER, H. P., HEILAND, S., PLATTEN, M., VON DEIMLING, A., VAN DEN BENT, M. J., GORLIA, T., WICK, W., BENDSZUS, M. & MAIER-HEIN, K. H. 2019. Automated quantitative tumour response assessment of MRI in neuro-oncology with artificial neural networks: a multicentre, retrospective study. *Lancet Oncology,* 20**,** 728-740.	Incorrect/missing outcome
KNOLLE, M., KAISSIS, G., JUNGMANN, F., ZIEGELMAYER, S., SASSE, D., MAKOWSKI, M., RUECKERT, D. & BRAREN, R. 2021. Efficient, high-performance semantic segmentation using multi-scale feature extraction. *PLoS ONE [Electronic Resource],* 16**,** e0255397.	Incorrect/missing outcome
KUMAR, G., SHARMA, N. & PAUL, A. 2023. An extremely lightweight CNN model for the diagnosis of chest radiographs in resource-constrained environments. *Medical Physics,* 50(12)**,** 7568-7578.	Incorrect/missing outcome
KUMAR, S., PAL, S., SINGH, V. P. & JAISWAL, P. 2023. Energy- efficient model "Inception V3 based on deep convolutional neural network" using cloud platform for detection of COVID-19 infected patients. *Epidemiologic Methods,* 12(1) .	Incorrect/missing outcome
KUMAR, S., SINGH, V. P., PAL, S. & JAISWAL, P. 2023. Energy-efficient model "denseNet201 based on deep convolutional neural network" using cloud platform for detection of COVID-19 infected patients. *Epidemiologic Methods,* 12(1) .	Incorrect/missing outcome
KUMAR, V. D., RAJESH, P., GEMAN, O., CRACIUN, M. D., ARIF, M. & FILIP, R. 2023. "Quo Vadis Diagnosis": Application of Informatics in Early Detection of Pneumothorax. *Diagnostics,* 13(7) .	Incorrect/missing outcome
LAI, Q., CHEN, W., DING, X., HUANG, X., JIANG, W., ZHANG, L., CHEN, J., GUO, D., ZHOU, Z. & CHEN, T. W. 2024. Quality control of elbow joint radiography using a YOLOv8-based artificial intelligence technology. *European Radiology Experimental,* 8(1) .	Incorrect/missing outcome
LANGIUS-WIFFEN, E., NIJHOLT, I. M., VAN DIJK, R. A., DE BOER, E., NIJBOER-OOSTERVELD, J., VELDHUIS, W. B., DE JONG, P. A. & BOOMSMA, M. F. 2024. An artificial intelligence algorithm for pulmonary embolism detection on polychromatic computed tomography: performance on virtual monochromatic images. *European Radiology,* 34**,** 384-390.	Incorrect/missing outcome
LEE, G. P., PARK, S. H., KIM, Y. J., CHUNG, J. W. & KIM, K. G. 2023. Enhancing Disease Classification in Abdominal CT Scans through RGB Superposition Methods and 2D Convolutional Neural Networks: A Study of Appendicitis and Diverticulitis. *Computational & Mathematical Methods in Medicine,* 2023**,** 7714483.	Incorrect/missing outcome
LESSAGE, X., LARHMAM, M. A., MAHMOUDI, S. & NEDJAR, I. 2018. Assessing breast cancer screening using recent deep convolutional neural networks. *International Journal of Computer Assisted Radiology and Surgery,* 13(Supplement 1)**,** S100-S102.	Incorrect/missing outcome
LI, J., CHOI, W. & ANNE, P. R. 2023. Deep-Learning Based Auto-Segmentation for Liver Yttrium-90 Selective Internal Radiation Therapy. *International Journal of Radiation Oncology Biology Physics,* 117(2 Supplement)**,** e684-e685.	Incorrect/missing outcome

LI, M., HSU, W., XIE, X., CONG, J. & GAO, W. 2020. SACNN: Self-Attention Convolutional Neural Network for Low-Dose CT Denoising with Self-Supervised Perceptual Loss Network. *IEEE Transactions on Medical Imaging,* 39(7)**,** 2289-2301.	Incorrect population
LI, N., ZHOU, S., WU, Z., ZHANG, B. & ZHAO, G. 2020. Statistical modeling and knowledge-based segmentation of cerebral artery based on TOF-MRA and MR-T1. *Computer Methods & Programs in Biomedicine,* 186**,** 105110.	Incorrect/missing outcome
LI, W., LU, H., WEN, Y., ZHOU, M., SHUAI, T., YOU, Y., ZHAO, J., LIAO, K., LU, C., LI, J., LI, Z., DIAO, K. & HE, Y. 2023. Reducing both radiation and contrast doses for overweight patients in coronary CT angiography with 80-kVp and deep learning image reconstruction. *European Journal of Radiology,* 161.	Incorrect/missing outcome
LI, Y., QIU, Z., FAN, X., LIU, X., CHANG, E. I. C. & XU, Y. 2022. Integrated 3d flow-based multi-atlas brain structure segmentation. *PLoS ONE [Electronic Resource],* 17(8 August) .	Incorrect/missing outcome
LI, Y. C., CHEN, H. H., HORNG-SHING LU, H., HONDAR WU, H. T., CHANG, M. C. & CHOU, P. H. 2021. Can a Deep-learning Model for the Automated Detection of Vertebral Fractures Approach the Performance Level of Human Subspecialists? *Clinical Orthopaedics & Related Research,* 479**,** 1598-1612.	Incorrect/missing outcome
LIU, F., GAO, L., WAN, J., LYU, Z. L., HUANG, Y. Y., LIU, C. & HAN, M. 2023. Recognition of Digital Dental X-ray Images Using a Convolutional Neural Network. *Journal of Digital Imaging,* 36**,** 73-79.	Incorrect/missing outcome
LIU, Y., HUANG, J., CHEN, J. C., CHEN, W., PAN, Y. & QIU, J. 2024. Predicting treatment response in multicenter non-small cell lung cancer patients based on federated learning. *BMC Cancer,* 24**,** 688.	Incorrect/missing outcome
LYU, T., ZHAO, W., ZHU, Y., WU, Z., ZHANG, Y., CHEN, Y., LUO, L., LI, S. & XING, L. 2021. Estimating dual-energy CT imaging from single-energy CT data with material decomposition convolutional neural network. *Medical Image Analysis,* 70**,** 102001.	Incorrect/missing outcome
MAJJI, R., G, O. P. P., RAJESWARI, R. & R, C. 2023. Smart IoT in Breast Cancer Detection Using Optimal Deep Learning. *Journal of Digital Imaging,* 36**,** 1489-1506.	Incorrect/missing outcome
MALIK, H., NAEEM, A., NAQVI, R. A. & LOH, W. K. 2023. DMFL_Net: A Federated Learning-Based Framework for the Classification of COVID-19 from Multiple Chest Diseases Using X-rays. *Sensors,* 23**,** 09.	Incorrect population
MAQSOOD, F., ZHENFEI, W., ALI, M. M., QIU, B., REHMAN, N. U., SABAH, F., MAHMOOD, T., DIN, I. & SARWAR, R. 2024. Artificial Intelligence-Based Classification of CT Images Using a Hybrid SpinalZFNet. *Interdisciplinary Sciences: Computational Life Sciences,* 16**,** 907-925.	Incorrect/missing outcome
MARIA JESI, P., KAMATCHI, S., MADHIVADHANI, D. & SARON GRACE, L. 2024. HRSHO: A hybrid rain optimized spotted hyena optimizer for efficient feature selection in CNN-based sinusitis classification. *Biomedical Signal Processing and Control,* Part B. 87.	Incorrect/missing outcome
MARTIN, S., ANDRE, R., TRABELSI, A., WOJAK, J., FORTANIER, E., ATTARIAN, S., GUYE, M., DUBOIS, M., ABDEDDAIM, R. & BENDAHAN, D. 2023. Compressed deep learning-based segmentation and fat fraction quantification in individual muscles of patients with muscle dystrophy. *Journal of Neuromuscular Diseases,* 10(Supplement 3)**,** S6.	Incorrect/missing outcome
MOSSA-BASHA, M., ZHU, C., PANDHI, T., MENDOZA, S., AZADBAKHT, J., SAFWAT, A., HOMEN, D., ZAMORA, C., GNANASEKARAN, D. K., PENG, R., CEN, S., DUDDALWAR, V., ALGER, J. R. & WANG, D. J. J. 2024. Deep Learning Denoising Improves CT Perfusion Image Quality in the Setting of Lower Contrast Dosing: A Feasibility Study. *Ajnr: American Journal of Neuroradiology,* 45**,** 1468-1474.	Incorrect population
MUHAMMAD, K., ULLAH, H., KHAN, Z. A., SAUDAGAR, A. K. J., ALTAMEEM, A., ALKHATHAMI, M., KHAN, M. B., ABUL HASANAT, M. H., MAHMOOD MALIK, K., HIJJI, M. & SAJJAD, M. 2022. WEENet:	Incorrect/missing outcome

An Intelligent System for Diagnosing COVID-19 and Lung Cancer in IoMT Environments. *Frontiers in Oncology,* 11.	
NASEER, A., YASIR, T., AZHAR, A., SHAKEEL, T. & ZAFAR, K. 2021. Computer-Aided Brain Tumor Diagnosis: Performance Evaluation of Deep Learner CNN Using Augmented Brain MRI. *International Journal of Biomedical Imaging,* 2021.	Incorrect/missing outcome
NOONAN, K. J., HEARNE, R. T., MURPHY, D. J., GAFFNEY, B., SHEEHY, B., MCVEIGH, N., FRANCIOSI, A. N., KEANE, M. P., ATAYA, A., GUPTA, N., MCCORMACK, F. X., BONELLA, F., BORIE, R., LYNCH, D. A., CURRRAN, K. & MCCARTHY, C. 2024. Evaluating the Performance of an Ensemble Neural Network Pipeline in the Classification of Chest CT Scans as Control, Diffuse Cystic Lung Disease and Emphysema. *American Journal of Respiratory and Critical Care Medicine. Conference: American Thoracic Society,* 209.	Incorrect/missing outcome
O'DONNELL, L. F., PIPIS, M., THORNTON, J. S., KANBER, B., WASTLING, S., MCDOWELL, A., ZAFEIROPOULOS, N., LAURA, M., SKORUPINSKA, M., RECORD, C. J., DOHERTY, C. M., HERRMANN, D. N., ZETTERBERG, H., HESLEGRAVE, A. J., LABAN, R., ROSSOR, A. M., MORROW, J. M. & REILLY, M. M. 2024. Quantitative MRI outcome measures in CMT1A using automated lower limb muscle segmentation. *Journal of Neurology, Neurosurgery & Psychiatry,* 95**,** 500-503.	Incorrect/missing outcome
PAPANDRIANOS, N., PAPAGEORGIOU, E. I., ANAGNOSTIS, A., FELEKI, A., PAPAGEORGIOU, K. & APOSTOLOPOULOS, D. J. 2020. Bone metastasis classification in bone scintigraphy using densely-connected convolutional neural networks. *European Journal of Nuclear Medicine and Molecular Imaging,* 47(SUPPL 1)**,** S406-S407.	Incorrect/missing outcome
PARK, S. H., KIM, Y. J., KIM, K. G., CHUNG, J. W., KIM, H. C., CHOI, I. Y., YOU, M. W., LEE, G. P. & HWANG, J. H. 2023. Comparison between single and serial computed tomography images in classification of acute appendicitis, acute right-sided diverticulitis, and normal appendix using EfficientNet. *PLoS ONE [Electronic Resource],* 18**,** e0281498.	Incorrect/missing outcome
POOJAR, P., QIAN, E., FERNANDES, T. T., NUNES, R. G., FUNG, M., QUARTERMAN, P., JAMBAWALIKAR, S. R., LIGNELLI, A. & GEETHANATH, S. 2023. Tailored magnetic resonance fingerprinting. *Magnetic Resonance Imaging,* 99**,** 81-90.	Incorrect/missing outcome
RAJAN, P. G. & SUNDAR, C. 2019. Brain Tumor Detection and Segmentation by Intensity Adjustment. *Journal of Medical Systems,* 43**,** 282.	Incorrect/missing outcome
REDDY, K. R. & DHULI, R. 2023. A Novel Lightweight CNN Architecture for the Diagnosis of Brain Tumors Using MR Images. *Diagnostics,* 13(2) .	Incorrect/missing outcome
REZAEI, Z., ESLAMI, B., KOMLEH, H. E. & JAHROMI, K. D. 2019. Abnormality detection in musculoskeletal radiographs by densenet and inception-v3. *Iranian Journal of Radiology,* Conference: 1st National Conference on Artificial Intelligence in Medical Imaging, AIMIN 2019. Tehran Iran, Islamic Republic of. 16**,** S14-S15.	Incorrect/missing outcome
ROACH, K. E., BIRD, A. L., PEDOIA, V., MAJUMDAR, S. & SOUZA, R. B. 2024. Automated evaluation of hip abductor muscle quality and size in hip osteoarthritis: Localized muscle regions are strongly associated with overall muscle quality. *Magnetic Resonance Imaging,* 111**,** 237-245.	Incorrect/missing outcome
SHAO, P. 2024. A novel 3D deep learning model to automatically demonstrate renal vasculature segmentation and its validation in nephron-sparing surgery. *European Urology,* 85(Supplement 1)**,** S2078.	Incorrect/missing outcome
SHIH, S. F., KAFALI, S. G., CALKINS, K. L. & WU, H. H. 2023. Uncertainty-aware physics-driven deep learning network for free-breathing liver fat and R<inf>2</inf>* quantification using self-gated stack-of-radial MRI. *Magnetic Resonance in Medicine,* 89(4)**,** 1567-1585.	Incorrect/missing outcome

SHIH, S. F., KAFALI, S. G., CALKINS, K. L. & WU, H. H. 2023. Uncertainty-aware physics-driven deep learning network for free-breathing liver fat and R₂ quantification using self-gated stack-of-radial MRI. *Magnetic Resonance in Medicine,* 89, 1567-1585.	Incorrect/missing outcome
SHU, X., YANG, Y. & WU, B. 2021. Adaptive segmentation model for liver CT images based on neural network and level set method. *Neurocomputing,* 453, 438-452.	Incorrect/missing outcome
SONIA, J. J. & DHEEBA, J. 2020. Classification of benign and malignant breast masses on mammograms for large datasets using core vector machines. *Current Medical Imaging Reviews,* 16(6), 703-710.	Incorrect/missing outcome
STANLEY, B. F. & WILFRED FRANKLIN, S. 2022. Automated cerebral microbleed detection using selective 3D gradient co-occurance matrix and convolutional neural network. *Biomedical Signal Processing and Control,* 75.	Incorrect/missing outcome
SU, K. H., KUO, J. W., JORDAN, D. W., VAN HEDENT, S., KLAHR, P., WEI, Z., AL HELO, R., LIANG, F., QIAN, P., PEREIRA, G. C., RASSOULI, N., GILKESON, R. C., TRAUGHBER, B. J., CHENG, C. W. & MUZIC, R. F. 2018. Machine learning-based dual-energy CT parametric mapping. *Physics in Medicine & Biology,* 63, 125001.	Incorrect/missing outcome
SUN, L., ZHAO, W., LYU, T., CHEN, Y., XING, L. & LIU, W. 2023. An Efficient Transformer Model for Synthesizing Dual Energy CT from Single Energy Scanner. *International Journal of Radiation Oncology Biology Physics,* 117(2 Supplement), e721-e722.	Incorrect/missing outcome
TAN, G. F. L., DU, T., LIU, J. S., CHAI, C. C., NYEIN, C. M. & LIU, A. Y. L. 2022. Automated lung ultrasound image assessment using artificial intelligence to identify fluid overload in dialysis patients. *BMC Nephrology,* 23, 410.	Incorrect/missing outcome
TANG, X., WAN CHAN TSEUNG, H., MOSELEY, D., ZVEROVITCH, A., HUGHES, C. O., GEORGE, J., JOHNSON, J. E., BREEN, W. G. & QIAN, J. 2024. Deep learning based linear energy transfer calculation for proton therapy. *Physics in Medicine & Biology,* 69, 30.	Incorrect population
TAPPEINER, E., PROLL, S., HONIG, M., RAUDASCHL, P. F., ZAFFINO, P., SPADEA, M. F., SHARP, G. C., SCHUBERT, R. & FRITSCHER, K. 2019. Multi-organ segmentation of the head and neck area: an efficient hierarchical neural networks approach. *International Journal of Computer Assisted Radiology & Surgery,* 14, 745-754.	Incorrect/missing outcome
TOSCANO, M., ARROYO, J., SAAVEDRA, A. C., MARINI, T. J., BARAN, T. M., DRENNAN, K., DOZIER, A. M., TAMAYO, L., ZHAO, T. & CASTANEDA, B. 2022. Testing a deep learning algorithm for automatic detection of prenatal ultrasound for under-resourced communities. *American Journal of Obstetrics and Gynecology,* 226(1 Supplement), S353-S354.	Incorrect/missing outcome
TUNG, C. H., LIU, C. K. & HUANG, H. M. 2023. Feasibility of Using Deep Learning to Generate Dual-Energy CT from 120-kV CT. *Journal of Medical and Biological Engineering,* 43(1), 93-101.	Incorrect/missing outcome
UEMURA, T., NAPPI, J., LU, H., KIM, H., TACHIBANA, R., HIRONAKA, T. & YOSHIDA, H. 2019. Reduction of false-positive polyp detections in CT colonography using multiscale 3D residual networks. *International Journal of Computer Assisted Radiology and Surgery,* 14(Supplement 1), S67-S68.	Incorrect/missing outcome
ULLAH, F., SRIVASTAVA, G., XIAO, H., ULLAH, S., LIN, J. C. W. & ZHAO, Y. 2024. A Scalable Federated Learning Approach for Collaborative Smart Healthcare Systems with Intermittent Clients Using Medical Imaging. *IEEE Journal of Biomedical and Health Informatics,* 28(6), 3293-3304.	Incorrect/missing outcome
UMAMAHESWARI, T. & BABU, Y. M. M. 2024. ViT-MAENB7: An innovative breast cancer diagnosis model from 3D mammograms using advanced segmentation and classification process. *Computer Methods and Programs in Biomedicine,* 257.	Incorrect/missing outcome

VAN DEN BORRE, I., PEIFFER, M., HUYSENTRUYT, R., HUYGHE, M., VERVELGHE, J., PIZURICA, A., AUDENAERT, E. A. & BURSSENS, A. 2024. Development and validation of a fully automated tool to quantify 3D foot and ankle alignment using weight-bearing CT. *Gait & Posture,* 113**,** 67-74.	Incorrect/missing outcome
VARALATCHOUMY, M. & RAVISHANKAR, M. 2020. Novel multiphase contouring and force calculation algorithm for ROI detection and calculation of energy value in multiple scale and orientation for early detection of stages of breast cancer. *International Journal of Medical Engineering and Informatics,* 12**,** 401-434.	Unable to find full text
VASQUEZ-VENEGAS, C., SOTOMAYOR, C. G., RAMOS, B., CASTANEDA, V., PEREIRA, G., CABRERA-VIVES, G. & HARTEL, S. 2024. Human-in-the-Loop-A Deep Learning Strategy in Combination with a Patient-Specific Gaussian Mixture Model Leads to the Fast Characterization of Volumetric Ground-Glass Opacity and Consolidation in the Computed Tomography Scans of COVID-19 Patients. *Journal of Clinical Medicine,* 13(17) .	Incorrect/missing outcome
VIJAYAKUMAR, S. R., AARTHY, S., DEEPA, D. & SURESH, P. 2025. Sustainable framework for automated segmentation and prediction of lung cancer in CT image using CapsNet with U-net segmentation. *Biomedical Signal Processing and Control,* 99.	Incorrect/missing outcome
VILLA, M., NASR, B., BENOIT, D., PADOY, N., VISVIKIS, D. & BERT, J. 2023. Fast dose calculation in x-ray guided interventions by using deep learning. *Physics in Medicine & Biology,* 68**,** 31.	Incorrect/missing outcome
WANG, R., CHEN, X., ZHANG, X., HE, P., MA, J., CUI, H., CAO, X., NIAN, Y., XU, X., WU, W. & WU, Y. 2024. Automatic segmentation of esophageal cancer, metastatic lymph nodes and their adjacent structures in CTA images based on the UperNet Swin network. *Cancer Medicine,* 13**,** e70188.	Incorrect/missing outcome
WANG, T., LEI, Y., HARMS, J., LIU, Y., GHAVIDEL, B., LIN, L., CURRAN, W., BEITLER, J., LIU, T., ZHOU, J. & YANG, X. 2019. Dual-energy CT-based accurate stopping power mapping using 3D deep convolutional neural networks for head-and-neck patients. *Medical Physics,* 46(6)**,** e233.	Incorrect/missing outcome
XIE, L., XU, Y., ZHENG, M., CHEN, Y., SUN, M., ARCHER, M. A., MAO, W., TONG, Y. & WAN, Y. 2024. An anthropomorphic diagnosis system of pulmonary nodules using weak annotation-based deep learning. *Computerized Medical Imaging and Graphics,* 118.	Incorrect/missing outcome
XIE, L., XU, Y., ZHENG, M., CHEN, Y., SUN, M., ARCHER, M. A., WAN, Y., MAO, W. & TONG, Y. 2024. An Anthropomorphic Diagnosis System of Pulmonary Nodules using Weak Annotation-Based Deep Learning. *medRxiv.,* 05.	Incorrect study type
XIE, M., GAO, J., ZHU, C. & ZHOU, Y. 2015. A modified method for MRF segmentation and bias correction of MR image with intensity inhomogeneity. *Medical & Biological Engineering & Computing,* 53**,** 23-35.	Incorrect/missing outcome
XING, Y., YANG, Z., LI, D. & NAI, W. 2020. Classification method of rectal cancer medical image based on ensemble learning of multiple deep artificial neural networks and filtering technology. *Basic and Clinical Pharmacology and Toxicology,* 127(SUPPL 3)**,** 35-36.	Incorrect/missing outcome
YAMAMOTO, N., SUKEGAWA, S., KITAMURA, A., GOTO, R., NODA, T., NAKANO, K., TAKABATAKE, K., KAWAI, H., NAGATSUKA, H., KAWASAKI, K., FURUKI, Y. & OZAKI, T. 2020. Deep Learning for Osteoporosis Classification Using Hip Radiographs and Patient Clinical Covariates. *Biomolecules,* 10**,** 10.	Incorrect/missing outcome
YOUSEFIRIZI, F. & RAHMIMR, A. 2021. Consolidating deep learning framework with active contour model for improved PET-CT segmentation. *Journal of Nuclear Medicine. Conference: Society of Nuclear Medicine and Molecular Imaging Annual Meeting, SNMMI,* 62.	Incorrect/missing outcome
YU, Y., XIAO, Y., CHENG, J. & CHIU, B. 2018. Breast lesion classification based on supersonic shear-wave elastography and automated lesion segmentation from B-mode ultrasound images. *Computers in Biology & Medicine,* 93**,** 31-46.	Incorrect/missing outcome

YUE, Y., BALTES, M., ABUHAJAR, N., SUN, T., KARANTH, A., SMITH, C. D., BIHL, T. & LIU, J. 2023. Spiking neural networks fine-tuning for brain image segmentation. *Frontiers in Neuroscience,* 17.	Incorrect/missing outcome
ZHANG, G., YANG, Z., HUO, B., CHAI, S. & JIANG, S. 2021. Automatic segmentation of organs at risk and tumors in CT images of lung cancer from partially labelled datasets with a semi-supervised conditional nnU-Net. *Computer Methods & Programs in Biomedicine,* 211**,** 106419.	Incorrect/missing outcome
ZHAO, H., LIU, Z., TANG, J., GAO, B., QIN, Q., LI, J., ZHOU, Y., YAO, P., XI, Y., LIN, Y., QIAN, H. & WU, H. 2023. Energy-efficient high-fidelity image reconstruction with memristor arrays for medical diagnosis. *Nature communications,* 14(1) .	Incorrect population
ZHAO, Y., TANG, C., CUI, B., SOMASUNDARAM, A., RASPE, J., HU, X., HOLZAPFEL, C., JUNKER, D., HAUNER, H., MENZE, B., WU, M. & KARAMPINOS, D. 2023. Automated segmentation of the human supraclavicular fat depot via deep neural network in water-fat separated magnetic resonance images. *Quantitative Imaging in Medicine and Surgery,* 13(7)**,** 4699-4715.	Incorrect/missing outcome
ZHU, H., LI, H., YAN, G., BUCH, S., WANG, Y., HAACKE, E. M., HUA, J. & ZHONG, Z. 2024. ROSE: Multi-level super-resolution-oriented semantic embedding for 3D microvasculature segmentation from low-resolution images. *Neurocomputing,* 599.	Incorrect/missing outcome
ZHU, X., ZHU, L., SONG, D., WANG, D., WU, F. & WU, J. 2023. Comparison of single- and dual-energy CT combined with artificial intelligence for the diagnosis of pulmonary nodules. *Clinical Radiology,* 78**,** e99-e105.	Incorrect/missing outcome
ZHUANG, X., LI, L., PAYER, C., STERN, D., URSCHLER, M., HEINRICH, M. P., OSTER, J., SMEDBY, O., BIAN, C., YANG, X., HENG, P. A., MORTAZI, A., BAGCI, U., SUN, C., GALISOT, G., RAMEL, J. Y., BROUARD, T., TONG, Q., SI, W., LIAO, X., ZENG, G., SHI, Z., ZHENG, G., WANG, C., MACGILLIVRAY, T., NEWBY, D., RHODE, K., OURSELIN, S., MOHIADDIN, R., KEEGAN, J., FIRMIN, D. & YANG, G. 2019. Evaluation of algorithms for Multi-Modality Whole Heart Segmentation: An open-access grand challenge. *Medical Image Analysis,* 58.	Incorrect/missing outcome
ZIMMERMANN, J. M., VICENTINI, L., VAN STORY, D., POZZOLI, A., TARAMASSO, M., LOHMEYER, Q., MAISANO, F. & MEBOLDT, M. 2020. Quantification of Avoidable Radiation Exposure in Interventional Fluoroscopy With Eye Tracking Technology. *Investigative Radiology,* 55**,** 457-462.	Incorrect/missing outcome
